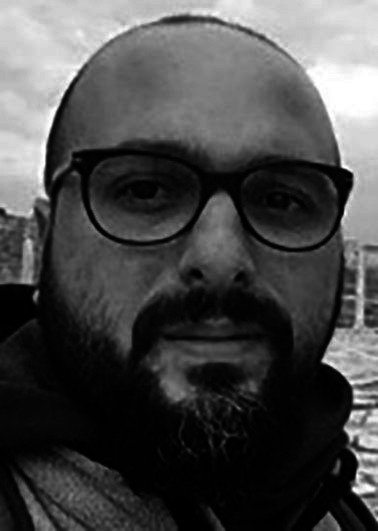# Recent Advances in Waste Plastic Transformation into Valuable Platinum‐Group Metal‐Free Electrocatalysts for Oxygen Reduction Reaction

**DOI:** 10.1002/cssc.202101252

**Published:** 2021-08-15

**Authors:** Mohsin Muhyuddin, Piercarlo Mustarelli, Carlo Santoro

**Affiliations:** ^1^ Department of Material Science University of Milano-Bicocca U5 Via Cozzi 55 20125 Milan Italy

**Keywords:** electrocatalysis, oxygen reduction reaction, plastics, surface chemistry, waste valorization

## Abstract

Plastic waste causes severe environmental hazards, owing to inadequate disposal and limited recycling. Under the framework of circular economy, there are urgent demands to valorize plastic waste more safely and sustainably. Therefore, much scientific interest has been witnessed recently in plastic waste‐derived electrocatalysts for the oxygen reduction reaction (ORR), where the plastic waste acts as a cost‐effective and easily available precursor for the carbon backbone. The ORR is not only a key efficiency indicator for fuel cells and metal–air batteries but also a major obstacle for their commercial realization. The applicability of the aforementioned electrochemical devices is limited, owing to sluggish ORR activity and expensive platinum‐group metal electrocatalysts. However, waste‐derived ORR electrocatalysts are emerging as a potential substitute that could be inexpensively fabricated upon the conversion of plastic waste into active materials containing earth‐abundant transition metals. In this Minireview, very recent research developments regarding plastic waste‐derived ORR electrocatalysts are critically summarized with a prime focus on the followed synthesis routes, physicochemical properties of the derived electrocatalysts, and their ultimate electrochemical performance. Finally, the prospects for the future development of plastic waste‐derived electrocatalysts are discussed.

## Graphical abstract

**From the waste up**: This Minireview concerns the transformation of waste plastic into valuable electrocatalysts for the oxygen reduction reaction. Transformations through controlled pyrolysis and microwave‐assisted techniques are described and further functionalizations are presented. The surface chemistry and morphology obtained after these processes are introduced and discussed, as is the activity of the plastic waste‐derived electrocatalysts.

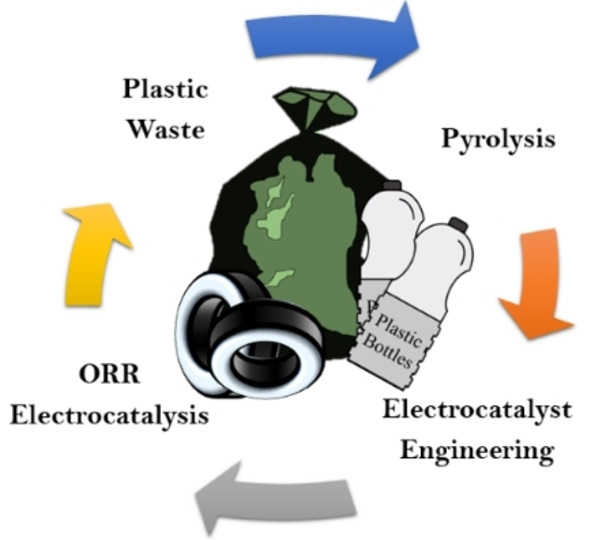

## Introduction

The unique combination of physicochemical properties and structural versatility offered by synthetic polymers make them one of the most diffuse classes of advanced materials. Currently, annual global production of plastics has surpassed the figure of 300 million tons,[^1^, ^2^, ^3^] of which 60 million tons are produced in Europe alone.[^4^, ^5^] Since the mid‐twentieth century, worldwide plastic manufacturing has been increasing at a yearly growth rate of approximately 8.5 % and it is estimated to reach the level of 500 million tons per year by the end of 2025.^[6]^ The overwhelming utility of plastics in domestic, commercial, and industrial applications cannot be denied; however, their service life is quite limited.^[5]^ It is estimated that nearly 60 % of plastics end up being part of waste streams,^[7]^ causing various environmental dilemmas because of their inadequate end‐of‐life (EoL) treatments.^[8]^ Plastic waste is not only responsible for land and soil pollution but also affects the aquatic environment, marine life and its ecology due to discharge into water bodies.^[9]^ The majority of oceanic plastics come from land‐based origins, which enter into marine domains by mismanaged dumping of sewage, coastal landfill, and litter carried through water channels.^[10]^ In addition to conventional plastic wastes, the disposal of scrap tires is also a persistent challenge.

It is estimated that by end of the year 2030, all‐inclusive scrap tire generation will reach 1.2 billion tires yearly,[^11^, ^12^, ^13^] whereas, currently an enormous magnitude of scrap tires nearly equal to 5 billion has already stockpiled that requires recycling treatments on the regular basis.[^13^, ^14^] Meanwhile, the total unprocessed polymeric waste gathered in the European Union (EU) is more than 27 million tons/year.[^4^, ^5^] It is important to underline that, previously, a significant portion of plastic waste was being exported to South Asia, but since these countries imposed restrictions on its import, hence, Europe must find out new ways to deal with this source.[^15^, ^16^]

Polymeric waste management in Europe relies on various routes and, according to the statistics, 31 % of waste is recycled, 42 % allows energy recovery through incineration, and the left 27 % of polymeric waste is still landfilled.[^4^, ^5^, ^17^] Contrarily to thermoplastics, thermosets and rubber tires are nearly impossible to recycle due to their crosslinks‐based high structural stability. As a result, they usually arrive either at landfill or become a part of energy recovery processes through conventional incinerating pathways, hence contributing to the planetary carbon footprint.^[18]^ Considering the aforesaid problems, the European Commission introduced a concept of sustainable management under the theme of “*A European Strategy for Plastics in a Circular Economy*”.[^5^, ^17^, ^19^] This strategy sheds light on the problems encountered during plastic waste management and proposes the conversion of plastic waste into useful products. It should be noted that the challenges are not only encountered in the volume of plastic waste being recycled, but the poor quality of resulting secondary plastic is also a big economic problem. In financial terms, more than 95 % of plastic waste – worth more than 100 billion euros – is lost every year.^[20]^ Consequently, a highly sustainable and commercially viable recycling strategy for waste plastics is urgently required. Out of various feasible strategies, the valorization of the polymeric waste into carbon‐based nanomaterials through pyrolysis could be the most promising approach,[^1^, ^3^, ^5^, ^8^] which may find their potential applications within the domain of energy conversion and storage. Such waste‐derived carbons (specifically from scrap tires) can be synthesized at a price as low as 0.06$/kg, which is significantly less than that of derived from biomass having a comparable yield.[^21^, ^22^, ^23^, ^24^] This makes them the most economical and sustainable source of carbon in the market.^[21]^

In addition to inadequate waste management, the second leading problem faced by the contemporary era is the severe energy crisis due to foreseen extinction of fossil fuels and the emission of greenhouse gases. Keeping this in mind, the EU has framed a definite set of policies towards a low carbon economy aiming to ensure an 80 % reduction in greenhouse gases by the year 2025, where industry and transportation sectors have to significantly cut off their emissions.^[25]^ Such scenarios encourage the development of advanced energy conversion and storage technologies such as photovoltaics, fuel cells, electrolyzers, supercapacitors, and batteries. Among the aforementioned technologies, fuel cells are glossing under the spotlight of current research due to their broad range of applications.[^26^, ^27^] Fuel cells offer high energy conversion efficiencies at practically negligible carbon discharge, which can potentially reduce the discrepancies between environmental concerns and growing energy demands.^[28]^ However, the commercial realization of fuel cells is still restricted by higher costs and limited performance. The overall efficiency of the fuel cell is governed by the oxygen reduction reaction (ORR) at the cathode which is a critical bottleneck due to its slow kinetics and high overpotential leading to lower power densities.^[29]^ To deal with sluggish ORR, platinum‐group metals (PGMs), which are scarce and very expensive, are used to fabricate state‐of‐the‐art electrocatalysts, accounting for nearly 30 % of the total cost of a full device.[^30^, ^31^] Moreover, the long‐term stability of Pt is restricted due to its inferior tolerance against carbon monoxide, methanol and other pollutants naturally present in the atmosphere.[^32^, ^33^] To address this issue, many efforts have been made to develop PGM‐free ORR electrocatalysts utilizing earth‐abundant metals by biomimicking or bioinspired approaches.[^34^, ^35^, ^36^] PGM‐free ORR electrocatalysts contain several different active sites with nitrogen and a given transition metal. It has been shown that the most efficient active sites are the ones containing transition metal (TM) atomically dispersed coordinated in nitrogen pyridinic environment in the form of TM−N_*x*_ with *x*=2, 3, or 4.[^37^, ^38^, ^39^, ^40^, ^41^, ^42^]

Carbon‐based PGM‐free electrocatalysts, specifically nitrogen‐doped,[^43^, ^44^] and iron‐nitrogen‐carbons (Fe−N−C),[^45^, ^46^, ^47^] have emerged as the most promising candidates to replace the PGM‐carrying electrocatalysts. In both scenarios, carbon provides a structural backbone to the catalysts. Furthermore, carbon is also employed as a support for the PGM‐based electrocatalysts which makes carbon support an essential research theme for ORR applications.^[48]^ Carbon‐based ORR electrocatalysts can be fabricated through the valorization of plastic waste in a facile and cost‐effective way.[^49^, ^50^, ^51^, ^52^] Hence, both ineffective waste management and energy crisis can be addressed simultaneously. Till now significant research has been conducted in the arena of waste‐derived ORR electrocatalysts but it is more inclined towards biomass waste.[^53^, ^54^, ^55^, ^56^] Plastic waste is more problematic due to its slow degradation; whereas, being composed of carbon as the main component, it can act as a widely available precursor for the fabrication of PGM‐free electrocatalysts in the most cost‐effective way. This Minireview groups all the recent advancements done in the synthesis of PGM‐free electrocatalysts, starting from waste plastic as an active precursor during the synthetic process. Emphasis is put on the possibility of transforming waste into a valuable product within the circular economy paradigm.

### Synthetic routes

This section is dedicated to discussing synthetic routes that have been pursued in the transformation of waste plastics into valuable electrocatalysts. By virtue of pyrolysis processes, carbon‐based nanomaterials can be easily produced through the utilization of waste plastics as a cost‐effective precursor.[^57^, ^58^] Pyrolysis is a thermal technique entirely conducted in controlled environments with the absence of oxygen and water. This process contrasts with other high‐temperature treatments, such as hydrolysis and combustion, where oxygen and water might be present.[^59^, ^60^] Generally, the three main products of pyrolysis are oil, carbonaceous char, and noncondensable gas.^[61]^ The oil generated can be refined and then used to run turbines, industrial furnaces, high‐duty engines, and boilers, whereas the char and noncondensable gas can act as a precursor for the fabrication of carbon‐containing ORR electrocatalysts.[^5^, ^62^] Recently, biomass waste‐derived ORR electrocatalysts have gained much attention, since biomass waste majorly comprises lignocellulose, carbohydrates, polysaccharides and animal biomass which produce relatively more carbon yield during pyrolysis and the obtained carbon‐based frameworks are relatively simpler to engineer.[^30^, ^53^, ^54^, ^55^, ^56^, ^59^] No doubt, obtaining higher solid carbon yield from the direct pyrolysis of the waste plastic is a technical issue since most of the mass transforms into oil‐based and noncondensable gaseous products.[^61^, ^63^, ^64^] However, by controlling the process parameter carbon yield could be enhanced, moreover, the obtained oil and noncondensable gas also contain important hydrocarbons of high economic value.[^5^, ^64^, ^65^]

**Figure 1:**
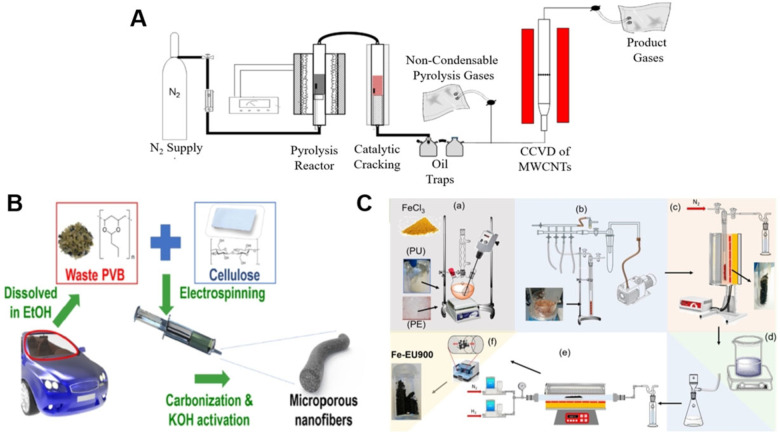
Different synthetic routes for ORR electrocatalysts. (A) Three‐stage process for the pyrolysis of flexible plastic packaging waste, catalytic cracking of oil, and MWCNTs deposition. Adapted with permission from Ref. [57]; copyright Elsevier 2020. (B) Synthesis of carbon nanofibers using cellulose and waste PVB. Adapted with permission from Ref. [49]; copyright Elsevier 2020. (C) Upcycling of waste polyurethane (PU) into iron and nitrogen‐codoped electrocatalyst through two‐step pyrolysis where the supplementary stages are as follows: a) mechanical mixing of polymers and FeCl_3_; b) drying and degassing of the polymer blend; c) double step pyrolysis; d) acid wash in H_2_SO_4_ at 100 °C and filtration on a nylon membrane; e) thermal activation in H_2_ flow; f) ball milling. Adapted from Ref. [51] under CC‐BY‐NC‐ND 4.0 (https://creativecommons.org/licenses/by‐nc‐nd/4.0).

Out of various plastics, polyethylene terephthalate (PET) is the most commonly applied packaging material, particularly for beverage containers and disposable water bottles, which contribute nearly 8 % of municipal solid waste.^[66]^ Above and beyond this, the annual consumption of PET is increasing with a constant rate of 6.0 wt% and making a serious environmental challenge due to its nonbiodegradability. In 2019, Veksha et al. reported a systematic transformation of PET‐containing waste plastic packaging into pyrolyzed oil and multi‐walled carbon nanotubes (MWCNTs) for ORR.^[57]^ Their pathway was comprising of three sequential steps; precisely, 1) pyrolysis of PET containing plastic mixture, 2) up‐gradation of condensed oil through catalytic cracking of vaporous hydrocarbons using 9.0 wt% Fe‐loaded zeolite catalyst, and 3) conversion of noncondensable gas into MWCNTs through catalytic chemical vapor deposition (CCVD) over 0.9 wt% Ni supported CaCO_3_ catalyst. The reaction temperatures for pyrolysis and CCVD were maintained at 400 °C and 700 °C, respectively, while the whole procedure was performed under nitrogen flux. The produced MWCNTs had to be washed with boiling HCl for 30 min, in order to remove the metallic impurities, such as Ni and Ca. The synthetic process is shown in Figure 1A. Since the composition of mixed plastic has a marked influence over the final yield of pyrolysis, Veksha et al. also analyzed two different compositions of plastic feedstocks containing 11.8 and 27.5 wt% PET, respectively. The yield of MWCNTs from plastic mixture with 11.8 wt% PET was higher than that of carrying 27.5 wt% PET. That observation could be related to a larger extent of carbon gasification due to CO_2_, and a smaller content of hydrocarbon in the noncondensable gas produced from the plastic mixture with a higher proportion of PET.^[67]^

Moo et al. further extended the research on plastic‐derived CNTs and analyzed the effects of plastic feedstock and synthesis temperature.^[68]^ They first pyrolyzed a blend of mixed plastics (MP) including 40 % low‐density polyethylene (LDPE), 40 % polypropylene (PP), 10 % polystyrene (PS), and 10 % PET at 600 °C. The noncondensable gas produced during pyrolysis was then converted into CNTs in the temperature range of 500–800 °C by CCVD. The operational temperature of CCVD was found to be an additional influencer on the final yield of CNTs, which was significantly improved as temperature increased from 500 °C to 800 °C.[^68^, ^69^] Recently, Sridhar and Park introduced microwave‐assisted transformation of waste PET bottles into three‐dimensional nitrogen‐doped CNTs with supplemented mesoporosity.^[70]^ For the synthesis of CNTs, a mixture containing urea, graphene oxide (GO), iron acetate, and finely cut waste PET in definite proportions was subjected to the exposure of microwaves for 5 min at 0.7 kW. Urea played the dual role of depolymerizing the waste PET and doping of nitrogen into carbon, while the iron nanoparticles (NPs) dispersed on the graphene substrate catalyzed the growth of CNTs in bamboo‐like shape. Employing this procedure, Sridhar and Park successfully ensured a relatively higher yield (ca. 22.9 wt%) of the CNTs.^[70]^

Polyvinyl butyral (PVB) is a flexible, optically clear and tough thermoplastic, commonly employed as a glass laminate in the windscreens of automobiles.^[71]^ Since a typical windscreen contains nearly 1 kilogram of PVB, 1.3 million tons per year of waste PVB is generated all over the world, which is still increasing due to a nonstop proliferation in the automotive industry.[^71^, ^72^] Recently, Park and coworkers formulated an innovative methodology to valorize the waste PVB together with environmentally‐friendly cellulose into metal‐free ORR electrocatalysts.^[49]^ The production route for the waste PVB‐derived nanofibers was based on three consecutive steps: a) electrospinning of a mixture containing cellulose acetate and waste PVB (in different weight ratios) at 12 kilovolts, b) carbonization at 800 °C in argon flux, and **c)** final activation with KOH (nanofibers to KOH ratio was 1 : 2) at 800 °C (Figure 1B). Here, electrospinning ensured the successful synthesis of unidimensional nanofibers with high structural uniformity; whereas, carbonization caused the formation of graphitic layers with various surface and edge defects.^[49]^ Finally, the decomposition of K_2_CO_3_ at 800 °C during KOH activation subsequently increased the microporosity.[^49^, ^73^] A good combination of structural defects and dispersed microporosity could help in achieving the goal of satisfactory ORR parameters.

PP is a saturated straight‐chain polymer that is extensively applied in many applications owing to its superior toughness and low density.^[61]^ As a result, left‐over PP is one of the largest contributors in the solid urban waste, constituting more than 24 % volume of the total plastic waste produced around the globe.[^61^, ^74^, ^75^] Cai et al. made a rational attempt to transform disposable PP lunchboxes into iron and nitrogen‐codoped CNTs through catalytic pyrolysis.^[52]^ They used a bi‐stage fixed‐bed reactor with two separate heating zones, where the upper stage was dedicated for the pyrolysis of waste PP feedstock and the lower stage contained iron‐loaded alumina catalysts, at the fixed temperature of 500 °C and 800 °C, respectively. The achieved iron‐doped CNTs (Fe−CNTs) were subsequently refluxed with nitric acid to remove the metallic impurities. To carry out nitrogen doping, Fe−CNTs were mixed with melamine (nitrogen precursor) and subjected to the second pyrolysis in the temperature range of 750–900 °C for 2 h in an argon environment. The iron and nitrogen‐codoped CNTs obtained at 850 °C during the second pyrolysis (Fe−N−CNT850) demonstrated outstanding ORR activity and stability compared to the benchmark electrocatalyst (20 wt% Pt/C). In recent times, it has been verified that by introducing a second metal doping (i. e. Ni, Mn, Co, etc.) into PGM‐free electrocatalysts, ORR characteristics can be synergistically enhanced.[^76^, ^77^] Ni is an inexpensive transition metal having electronic characteristics similar to that of Fe,^[78]^ and resembling Pt or Pd in terms of reactivity as proven through its employment for ORR.^[79]^ Inspired from theses merits of Ni, Cai et al. reported a mechanistic study to develop Fe−Ni‐codoped carbon electrocatalyst derived from waste lunchboxes (PP).^[50]^ Catalytic vapour deposition (CVD) technique comprising a two‐stage fixed‐bed reactor was employed to produce oxidized CNTs with varying Fe/Ni ratios.

Polyurethanes (PU) are a major class of thermosetting polymers based on nitrogen‐rich urethane groups [−NH−(C=O)−O−]. Due to massive commercial demands, PU makes nearly 8.0 % of the total plastics produced and is positioned at 6^th^ place in the most utilized plastics around the world.^[80]^ On the other hand, nondegradable waste PU is causing serious problems due to its difficult recycling and eventual disposal into landfills.^[81]^ Latterly, Daniel et al. plotted a persuasive idea of upcycling waste PU into highly efficient iron and nitrogen‐codoped carbon as an electrocatalyst for ORR (Figure 1C).^[51]^ Firstly, they prepared a solution of low‐density polyethylene (LDPE), PU, and FeCl_3_ in p‐xylene solvent, where the LDPE was acting as a composite matrix, PU as a dual source of carbon and nitrogen, FeCl_3_ as the iron source and pore‐forming agent, and p‐xylene as a dispersant for homogenous mixing. The obtained mixture after the environment‐controlled drying was first pyrolyzed at 500 °C and then fluffy carbon was carefully separated from resultant wax‐like yellowish matter. In the next step, only the carbonaceous matter was subjected to the second pyrolysis at higher temperatures.^[51]^ Two‐steps pyrolysis is always advantageous to obtain a high yield of char from the plastic waste, whereas direct high‐temperature pyrolysis leads to continuous formation of liquids that could interfere with the process, drastically diminishing the carbon yield.^[5]^ The electrocatalyst achieved after the second pyrolysis was washed with 0.5 m H_2_SO_4_ and subsequently subjected to the third pyrolysis at 900 °C for 2 h in a reductive atmosphere (8.0 wt% H_2_ balanced with N_2_), which could regenerate and atomically disperse the Fe−N_*x*_ active sites in the carbon matrix.^[51]^

**Figure 2:**
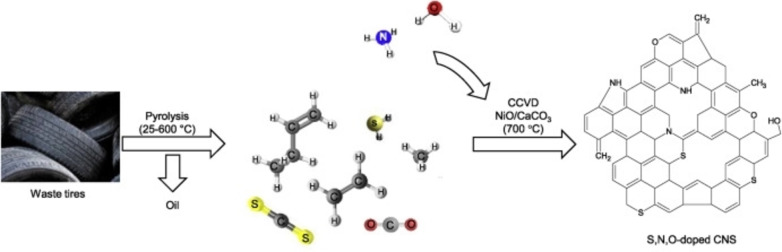
Synthesis of heteroatom‐doped carbon nanosheets (CNSs) derived from waste tires and their theoretical structure. Figure 2 adapted with permission from Ref. [82]; copyright Elsevier 2020.

From the time when the scrap tires began to enter waste streams, causing serious environmental hazards, an increased interest has emerged to treat scrap tires in an ecologically beneficial way.^[83]^ In recent times, González‐González et al. produced pyrolytic carbon‐black from the scrap tires using a combination of direct pyrolysis and chemical activation (using H_2_SO_4_ and KOH) as a potential candidate for nanomaterial precursor.^[84]^ Inspired by the extraordinary characteristics offered by the scrap tire‐derived carbon nanomaterials, Hood et al. first time reported the synthesis of carbon support for Pt‐based ORR electrocatalysts.^[21]^ They first heated tire crumbs at 400 °C to extract liquid oil and resulting carbon was then pyrolyzed at 1100 °C, which was finally activated with KOH to get a good combination of high surface‐area, dispersed porosity, sufficient crystallinity together with the possibility of higher Pt loading. Passaponti et al. pioneered the transformation of scrap tires into economical PGM‐free ORR electrocatalysts through simple microwave‐assisted pyrolysis (MAP) and analyzed the effect of annealing treatment under static air.^[85]^ In another study, carbonaceous char achieved from MAP of scrap tires was enriched with copper and cobalt through electrochemical and sonochemical deposition by taking advantage of the relaxed working conditions in terms of atmospheric pressure, room temperature and process simplicity.^[86]^ Very recently, a group of Korean researchers reported the synthesis of nitrogen‐doped, carbon‐based electrocatalysts resulting from the sulfonation and subsequent pyrolysis at 900 °C in NH_3_/Ar environment, where NH_3_ was acting as the nitrogen precursor.^[87]^ The superficial oxygen and sulfonyl functional groups introduced during sulfonation promoted successful nitrogen doping in the second step.^[87]^ Using a similar technique, Veksha et al. produced heteroatom‐doped electrocatalyst from scrap tires.^[82]^ The synthesis process was again based on the CCVD (Figure 2). However, before the employment of CCVD, noncondensable gas from the pyrolysis of scrap tires was saturated with water vapors and ammonia at room temperature to carry out nitrogen and oxygen doping, whereas the scrap tires acted as a precursor for sulfur and carbon. Table 1 provides a concise summary of different waste sources and applied strategies to achieved ORR electrocatalysts.

**Table 1:** ORR electrocatalysts obtained from different plastic waste sources.

Waste Plastic Source	Secondary Precursor/ Deposition Catalyst*	Synthesis route	Derived electrocatalyst	Optimal synthesis temperature [°C]	Acid wash	Ref.
PET packaging	NiO−CaCO_3_*	Pyrolysis, CCVD	Carbon‐based metal‐free	700	3 m HCl	[57]
Mixed plastics	NiO−CaCO_3_*	Pyrolysis, CCVD	Carbon‐based metal‐free	500	3 m HCl	[68]
PVB	Cellulose	Electrospinning, carbonization, KOH activation	Carbon‐based metal‐free	800	6 m HCl	[49]
PET bottles	Urea, GO, iron acetate	Microwave Irradiation	N‐doped carbon with Fe NPs	N/A	N/A	[70]
PP lunchboxes	Fe−Al_2_O_3_*, melamine	Catalytic pyrolysis	Fe,N‐codoped	850	20 wt% HNO_3_	[52]
PP lunchboxes	Fe−Ni−Al_2_O_3_*	Catalytic pyrolysis, CVD	Bimetallic doped	500	20 wt% HNO_3_	[50]
Polyurethane	LDPE, FeCl_3_ ⋅ 6H_2_O	Pyrolysis	Fe,N‐codoped	900	0.5 m H_2_SO_4_	[51]
Scrap tires	N/A	MAP, annealing	Carbon‐based metal‐free	450	(1 : 1) 98 % H_2_SO_4_ 70 % HNO_3_	[85]
Scrap tires	Co^II^ in NH_4_OH/HClO_4_ buffer solution	MAP, electrochemical deposition	Carbon enriched with Co	N/A	N/A	[86]
Scrap tires	NH_3_/Ar (5 : 5 v/v)	Sulfonation, pyrolysis	N‐doped carbon	900	98 % H_2_SO_4_	[87]
Scrap tires	NiO−CaCO_3_*, NH_3_, H_2_O vapors	Pyrolysis, CCVD	Heteroatom‐doped carbon	700	3 m HCl	[82]

### Surface chemistry and morphology

A set of descriptors for ORR activity must be defined because the catalytic activity of the electrocatalyst is governed by nature, plurality, atomic dispersion, and accessibility of active sites.[^47^, ^88^, ^89^, ^90^] Recently, two reactivity descriptors have been widely used: the active metal site density (SD [mol_site_ cm^−2^], SD_mass_ [site g^−1^_cat_], or SD_vol_ [site cm^−3^])^[91]^ and the catalytic turnover frequency (TOF [s^−1^] or [electron site^−1^ s^−1^]),^[92]^ which represent the electrons transferred per active site per second. SD and TOF and their relationships were deeply described in recent works.[^91^, ^92^]

Carbon‐based electrocatalysts exhibit several active moieties that could contribute to ORR. Depending upon nature, these moieties can be broadly classified into three main categories:[^88^, ^89^] 1) Structural defects and discontinuities in the main carbonaceous architecture of the electrocatalysts;[^44^, ^93^] 2) nitrogen‐containing active sites;^[94]^ 3) atomically dispersed MN_*x*_ active centers.[^40^, ^95^] Carbon‐based nanomaterials show two main defective regimes: basal planes, comprising 2D conjugated atoms with sp^2^ hybridization, and edge defects, containing unsaturated carbon atoms with dangling bonds along with oxygen‐carrying surface functionalities. The edge defects play an essential role in the heterogeneous electron transfer from the electrode to the electrolyte and hence could improve the electrocatalytic activity on the redox probe.[^44^, ^93^] Using Raman spectroscopy, such structural defects can be easily evaluated.^[96]^ In a typical Raman spectrum, the D band ($\bar{\nu }$ ≈1340 cm^−1^) appears due to sp^3^‐like edge defects and structural discontinuities; whereas the G band ($\bar{\nu }$ ≈1571 cm^−1^) is linked with vibrations of sp^2^‐hybridized atoms in the graphitic structure. The intensity ratio of the D and G band’ (*I*_D_/*I*_G_) can characteristically indicate any variations in the defect density and graphitic content of carbon.[^93^, ^96^] Catalytic reduction of oxygen and ORR intermediates on the pristine structure of carbon is still limited, usually it leads to production of peroxide and needs to be further regulated through heterogeneous doping.^[44]^ Doping of nitrogen into the sp^2^‐hybridized carbonaceous architecture is believed to be one of the most important strategies to modulate the ORR activity, by boosting the electron transfer rate because of increased electron density near the carbon Fermi level.[^28^, ^44^, ^54^] Higher electronegativity of nitrogen entails the charge redistribution and makes the adjacent carbon atoms more electropositive, resulting in better interaction with O_2_ for dissociative adsorption. In the electrocatalysts obtained from pyrolysis, a series of nitrogen‐containing moieties are formed and the catalytic performance towards ORR activity is improved, despite, due to the lack of the active site containing the transition metal, peroxide is generally formed. Particularly, pyridinic nitrogen is of interest because it is suspected to be an active site for the disproportion of the intermediate to the final reaction product H_2_O or OH^−^ (depending from the electrolyte pH operations).[^44^, ^88^]

Lastly, in the PGM‐free electrocatalysts, the moieties containing transition‐metal such as Fe, Co, Ni, Mn and Cu having multiple coordination with pyridinic nitrogen in the carbon matrix stimulate the ORR activity towards a four electron transfer mechanism.[^48^, ^88^, ^89^] For the investigation of these moieties, state‐of‐the‐art spectroscopic and microscopic characterization techniques can be used, such as X‐rays Photoelectron Spectroscopy (XPS), Mossbauer Spectroscopy (up to Fe element), and Aberration Corrected‐Scanning Transmission Electron Microscopy (AC‐STEM).[^88^, ^90^] Moreover, recently, also spectroscopic tools through synchrotron light have been widely adopted for identified the metal‐containing active sites.[^39^, ^40^] In addition to engineering and downsizing of active sites, the ORR activity is also dependent upon their accessibility, as the inaccessible moieties don't contribute actively to ORR. Therefore, a good combination of macropores (ion‐buffering pools), mesopores (channels for the rapid transportation of ions) and micropores (where the active sites are located) is essential.^[30]^ The surface area and distribution of porosity can be investigated through nitrogen and helium absorption isotherms with subsequent Brunauer‐Emmett‐Teller (BET) and Barrett‐Joyner‐Halenda (BJH) analyses.

The aforementioned ORR performance descriptors could be tailored through the optimization of synthesis parameters, such as choice of raw material and precursors, doping agents, pyrolyzing temperature, pyrolysis atmosphere, dwell time, and acid/base washing. Bearing this fact in mind, it is mandatory to fully understand the effects of these parameters (discussed in the previous sections) on the structural variation, surface chemistry, and morphological details, together with their ultimate impact on the overall efficiency of the synthesized electrocatalysts towards the ORR activity.

Veksha et al. reported the effect of PET incorporation in plastic feedstock on the structural and surface properties of the produced MWCNTs.^[57]^ It was observed that MWCNTs developed from the plastic feedstock with a lesser fraction of PET (11.8 wt%) had greater defect density and lower extent of graphitization as compared to those prepared from larger PET content (27.5 wt%). However, larger PET content caused an increase in the BET surface in parallel with a decrease in the surface oxygen functionalities. While analyzing the effect of precursor type and synthesis temperature, Moo et al. found that well‐defined CNTs were produced when LDPE and PP were used as a feedstock; however, the incorporation of PET in the plastic mixture produced important changes in the morphology creating additional nanocages.^[68]^ Moreover, the CNTs produced at 800 °C had a larger outer diameter than those produced at a lower temperature (500 °C). The synthesis temperature also influenced the structural characteristics and surface chemistry of the synthesized CNTs. Higher deposition temperature not only drastically decreased *I*_D_/*I*_G_ from 1.47 to 0.39 due to self‐annealing and graphitization in CNTs but also caused a significant increase in surface oxidation.^[68]^ Park and coworkers analyzed the effect of utilizing waste PVB in the cellulose matrix for the fabrication of nanofibers.^[49]^ Interestingly, the nanofibers derived from the mixture of waste PVB and cellulose showed superior morphological and textural properties over those prepared from pure cellulose. Waste‐PVB served a dual role during the carbonization at 800 °C, where the elimination of volatiles from the carbonaceous matrix of PVB introduced well‐dispersed microporosity, and the remaining PVB advantageously produced graphitic layers decorated with numerous surface defects. Hence, BET surface area of 698.1 m^2^ g^−1^ (pore volume of 0.2919 cm^3^ g^−1^) and Raman's ID/IG ratio of 1.00 synergically enhanced ORR activity of resultant nanofibers.^[49]^

As mentioned before, the nitrogen species remarkably boost up O_2_ adsorption and its successive reduction at the electrode surface.^[54]^ By deconvolution, the N1s peak of the XPS survey spectra depicts different types of nitrogen‐bonding configurations.^[97]^ Moreover, as often the quantity of transition metal is below 1 at%, this is not detected using XPS and therefore N1s peak is also used for quantifying the TM−N_*x*_ (TM as metal and *x*=2–4) contained on the surface of the electrocatalyst.

**Figure 3:**
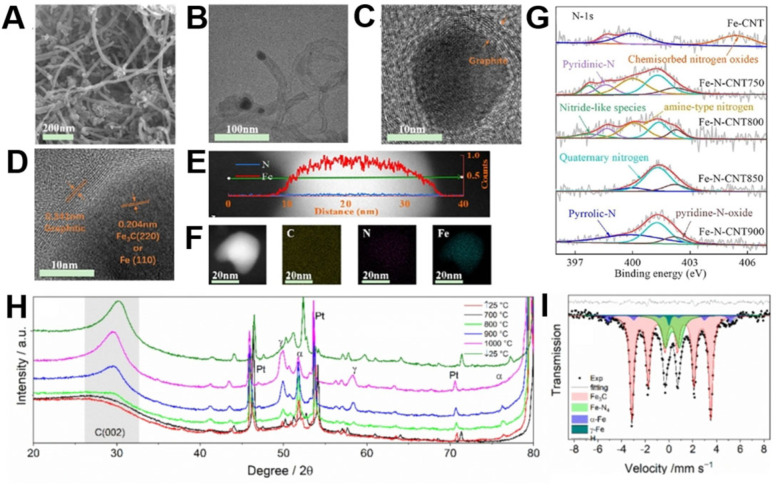
(A) SEM image of Fe−N−CNT850. (B) TEM image of Fe−N−CNT850 with encapsulated Fe nanoparticles. (C) HR‐TEM image showing the presence of Fe nanoparticles. (D) HR‐TEM image showing the presence of Fe_3_C. (E) High‐angle annular dark‐field scanning transmission electron microscopy (HAADF‐STEM) cross‐sectional compositional profiles. (F) HAADF‐STEM images with elemental mapping of C, N, and Fe. (G) High‐resolution XPS survey spectrum of N1s for Fe−N−CNT850, comparing the formation of various nitrogen species at different pyrolysis temperatures. Adapted from Ref. [52]; copyright Wiley‐VCH 2020. (H) X‐ray diffraction (XRD) characterization using a heating chamber and (I) Mössbauer spectra and Lorentzian fitting curves for waste PU‐derived electrocatalysts indicating temperature‐dependent transformations of the iron‐based moieties. Adapted from Ref. [51] under CC‐BY‐NC‐ND 4.0 (https://creativecommons.org/licenses/by‐nc‐nd/4.0).

Both synthesis strategy and choice of precursor affect the chemical structure and proportion of the nitrogen moieties in the resultant electrocatalysts, which differently endow the ORR mechanism.^[47]^ From the quantitative analysis of XPS survey scan of waste PET and urea‐derived CNTs, Sridhar and Park observed that nitrogen‐carrying moieties were mainly present in their pyridinic and pyrrolic configurations with relative distribution of 39 % and 61 %, respectively.^[70]^ Following the matching method of heterogeneous doping in the CNTs to uplift the ORR performance, Cai et al developed Fe−N−CNTs and analyzed the effect of second pyrolysis temperature.^[52]^ SEM micrographs confirmed the formation of well‐defined CNTs while the TEM images of prepared Fe−N−CNT at 850 °C demonstrated the encapsulation of nearly 10 nm iron particles between the graphitic layers of CNTs having an outer diameter in the range of 16 nm (Figure 3A–F).

Through XPS analysis, an obvious difference in the nitrogen species was observed for varying nitrogen‐doping temperatures (Figure 3G).^[52]^ Between the temperature range of 700 °C and 800 °C nitride‐like species were formed mainly due to the evolution of g‐CN_*x*_ during the thermal degradation of nitrogen precursor.[^52^, ^98^] As the nitrogen‐doping temperature increased further nitride‐like less stable species started to diminish and were completely converted into quaternary‐nitrogen at 850 °C through temperature‐assisted condensation reactions; whereas, the pyridinic nitrogen ultimately appeared at 900 °C.[^52^, ^99^] Nitrogen‐doping temperature also affected the structural characteristics of the catalysts, as the calculated ID/IG ratio tended to reduce with respect to an increase in temperature. This observation confirmed the temperature‐assisted microstructural rearrangements, leading to enhanced graphitization together with healing of structural defects.^[100]^

In a separate investigation into bimetallic ORR electrocatalysts derived from waste lunchboxes, Cai et al. experienced a clear influence of Fe and Ni ratios on the physicochemical properties of the produced CNTs.^[50]^ In comparison with Fe‐rich CNTs, higher Ni to Fe ratios promoted the formation of longer and thinner CNTs with relatively higher hydrophilic oxygen‐carrying surface groups, together with comparable ID/IG Raman ratios. However, an opposite trend was observed for the BET surface area and density of mesoporosity, where the highest BET surface was achieved when the Fe to Ni ratio was 2 : 1.^[50]^

Daniel et al. systematically analyzed the temperature‐dependent phase transformations during pyrolysis of iron and nitrogen‐based moieties in waste PU‐derived electrocatalysts.^[51]^ From in‐situ X‐rays diffraction (XRD), it was elucidated that as the pyrolysis temperature increased from 700 °C to 1000 °C, iron underwent phase transformations from Fe_3_C and α‐Fe to γ‐Fe, which sustained its structural configuration as a metastable state even after quenching to room temperature (Figure 3H). Further insights into iron‐based species through Mössbauer spectroscopy confirmed the formation of different sorts of well‐dispersed Fe‐containing active sites at 800 °C (Figure 3I),^[51]^ which are primarily important for the efficient ORR activity.^[89]^ Recently, the iron‐based single‐atom electrocatalysts (SAECs) have emerged as a new frontier in the domain of Fe−N−C ORR electrocatalysis, where structural heterogeneity and adjacent coordination improve the catalytic performance.[^101^, ^102^] Iron exhibits several empty d‐orbitals and allows various kinds of coordination, hence different types of iron‐containing monoatomic sites (Fe−N_4_, Fe−N_3_ and Fe−N_2_) are formed.[^88^, ^101^] Another interesting feature communicated by Daniel et al. was variation in the porosity distribution as a function of feedstock composition and pyrolysis temperature.^[51]^ It was revealed that the addition of LDPE restricted the nucleation of micropores and the formation of sponge‐like nonporous structures, whereas, microporosity appeared to increase with temperature. These two factors got synergized and brought about the highest ORR performance shown by the electrocatalyst fabricated from PU and FeCl_3_ at 900 °C without the involvement of LDPE.^[103]^

**Figure 4:**
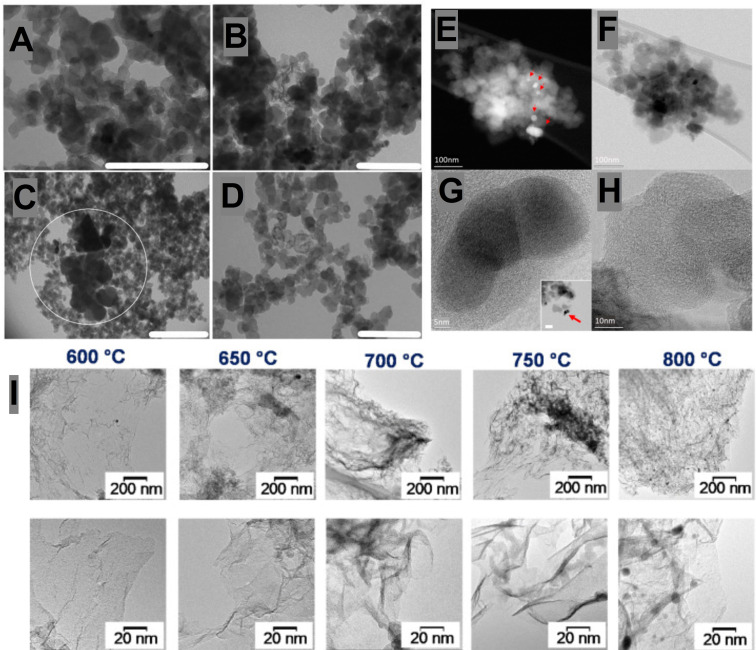
(A–D) TEM images of treated samples at different temperatures: A) 150°C; B) 300°C; C,D) 450°C. Adapted with permission from Ref. [85]; copyright Elsevier 2020. (E,F) High angle annular dark‐field (HAADF; E) and bright‐field (BF; F) STEM images of clusters of raw char sample typically at the sub‐micron scale. The raw char is crushed powder supported by amorphous carbon film. In HAADF STEM images, the Z contract shows inorganic nanocrystals which are indicated by the red arrows. (G,H) HR‐BF STEM images showing the crystalline nature of the nanoparticles, with a good degree of graphitization of the char material. Inset of (G) shows a magnified overview of the flake containing the nanoparticles. Adapted with permission from Ref. [86] under CC‐BY‐NC‐ND 4.0 (https://creativecommons.org/licenses/by‐nc‐nd/4.0); MDPI 2020. (I) TEM images of carbon materials produced at different temperatures using NiO‐loaded CaCO_3_ catalysts, taken at low and high resolutions (top and bottom, respectively). Adapted with permission from Ref. [82]; copyright Elsevier, 2020.

Considering the fabrication of ORR electrocatalysts from scrap tires, Passaponti et al. established a direct relationship between the synthesis conditions and the properties of the achieved electrocatalysts.^[85]^ Using TEM, nanoscale morphological surface features of the electrocatalysts treated at 150, 300 and 400 °C were investigated and the corresponding micrographs are shown in Figure 4A–D. All the samples demonstrated typical agglomerates of carbon particles with sizes in the range of 30–50 nm; however, the sample treated at 450 °C exhibited large clusters of ZnO NPs. BET surface area and microporosity came out to increase with annealing temperature under static air; however, the yield was quenched down by 97 % at 600 °C. Moreover, the by‐default impurity phase of β‐ZnS (remnants of the vulcanization treatment) in tires transformed into evenly dispersed ZnO nanocrystals in the matrix of carbon due to its reaction with atmospheric oxygen.^[85]^

The electrochemical deposition of cobalt on the char achieved from MAP of scrap tires provided a uniform network‐like coverage of cobalt, where the deposition time was postulated as a controlling parameter to optimize the coverage of NPs (Figure 4E–H).^[86]^ Kang et al. observed that sulfonation of scrap tires with sulfuric acid before pyrolysis initiated the formation of amorphous carbon, where the nitrogen content (4.3 at.%) was much higher than that of the nonsulfonated one (2.7 at%).^[87]^ This strategy significantly increased the BET surface area (493.8 m^2^ g^−1^) along with the coexistence of micro and mesopores of variable sizes. Veksha et al. demonstrated the growth of heteroatom‐doped two‐dimensional porous carbon‐nanosheets (CNSs) through the pyrolysis‐assisted CCVD of scrap tires.^[82]^ Up till 750 °C development of typical carbon nanosheets (CNSs) was predominant, however at 800 °C dense spherical NPs were additionally witnessed in the TEM micrographs, indicating the encapsulation of catalyst into the carbon matrix at elevated CCVD temperature (Figure 4I). Saturation of noncondensable pyrolysis gas with NH_3_ and water vapors led to a high BET surface area of 588 m^2^ g^−1^ and increased nitrogen content of 0.5 at% with a sufficient proportion of pyridinic nitrogen. Employment of saturation step had a negative impact on sulfur which was considerable reduced after nitrogen doping, though the oxygen surface functionalities were increased.^[82]^ The impacts of synthesis process parameters on the physicochemical properties of polymeric waste‐derived ORR electrocatalysts are summarized in Table 2.

**Table 2:** Physicochemical properties of plastic waste‐derived ORR electrocatalysts.

Waste plastic source	Morphology^[a]^	Surface area [m^2^ g^−1^]^[b]^	Pore volume [cm^3^ g^−1^]^[b]^	*I*_D_/*I*_G_^[c]^	Dopant atom/ surface functionality^[d]^	Ref.
PET packaging	MWCNTs	197	N/A	1.6	O: 8.9 at%	[57]
Mixed plastics	CNTs	N/A	N/A	1.47	O: 7 %	[68]
PVB	Nanofibers	698.1	0.2919	1.00	N/A	[49]
PET bottles	Bamboo‐like CNTs	N/A	N/A	1.36	N: 4.56, O: 1.17, Fe: 6.8 wt%	[70]
PP lunchboxes	CNTs	114.4	0.71	0.75	N: 0.69, Fe: 0.84 wt%	[52]
PP lunchboxes	CNTs	134.6	0.64	0.72	O: 18.33, Fe: 0.02, Ni: 0.06 mass	[50]
Polyurethane	Alveoli‐like structure	479	0.283	1.4	N: 1.2, N−O: 4.9, Fe: 0.1 at%	[51]
Scrap tires	Agglomerated carbon NPs	296	1.03	N/A	N/A	[85]
Scrap tires	Network‐like Co coverage on carbon NPs	N/A	N/A	N/A	N/A	[86]
Scrap tires	Amorphous carbon structure	493.8	0.50	N/A	N: 4.3, O: 4.1, S: 0.2 at%	[87]
Scrap tires	Nanosheets	588	N/A	0.94	N: 0.5, S: 0.4, O: 6.8 at%	[82]

[a] From TEM/SEM. [b] From BET. [c] From Raman spectroscopy. [d] From XPS survey spectra.

### Electrochemical tests and results

Electrocatalytic reduction of oxygen is a multi‐electron transfer mechanism consisting of various elementary stages of huge complexity. The reaction mechanism of each underlying step is fundamentally governed by the pH of the electrolyte.[^53^, ^54^, ^104^] Depending upon the nature of electrolyte employed, the two important classes of electrochemical fuel cells are proton exchange membrane fuel cells (PEMFCs) and anion exchange membrane fuel cells (AEMFCs), offering several advantages over one another.[^32^, ^48^, ^105^, ^106^] The scope of this Minireview is limited to the very recent research developments in the domain of plastic waste‐derived ORR electrocatalysts. Existing literature related to ORR mechanisms and reaction pathways^[107—^, ^110]^ and electrocatalysts[^36^, ^88^, ^111^, ^112^, ^113^, ^114^, ^115^] is reported for acidic media and alkaline media for PEMFCs and AEMFCs, respectively. As mentioned before, the electronic pathway of ORR reaction differs at different pH of the electrolyte.^[116]^ For instance, in the acidic medium, protons (H^+^) are combined with oxygen at the cathodic site of the fuel cell, which undergoes various alternative reduction pathways. ORR either follows a two‐electrons pathway where the end product is H_2_O_2_ or a four‐electrons pathway with water as a final product,^[104]^ whereas a consecutive 2×2e^−^ pathway causes the reduction of H_2_O_2_ at the same or different catalytically active sites with the generation of H_2_O.^[117]^ The hydrogen peroxide generated in the earlier step of ORR is converted into water by chemical decomposition. The electrocatalytic contribution of nitrogen and surface metallic moieties for all the afore‐explained three ORR mechanisms has been studied by Georgescu et al.^[118]^ Likewise, in the alkaline pH, OH^−^ radicals play a vital role in ORR as the main product (during direct four‐electrons pathway) where the OH_2_^−^ are generated during the two‐electrons reduction mechanism.^[104]^ Stepwise 2×2e^−^ mechanism can also take place in the alkaline media. Kinetic investigations suggest that the best electrocatalysts drive the ORR activity with a greater contribution of the tetra‐electronic pathway, leading to enhanced current densities, operating potentials and fewer intermediate peroxide species production.^[54]^ In order to evaluate the efficacy of an electrocatalyst towards ORR activity and its kinetic parameters, a ring disk electrode (RDE) or rotating‐ring disk electrode (RRDE) coupled with cyclic voltammetry (CV) is commonly employed as a fast probe. RDE is a hydrodynamic electrode system consisting of a disk and a rotating shaft, whereas RRDE has an extra coaxial Pt ring electrode for the detection of products generated from the disk electrode. Readers may study the previously published literature regarding the utilization of RDE and RRDE from the point of view of practical ORR electrochemical evaluations.[^32^, ^109^, ^119^] Using RDE/RRDE, linear‐scan voltammograms of the ring current and disk current densities versus potential are recorded, where the performance indicators are onset‐potential (*E*_onset_), half wave‐potential (*E*_1/2_), limiting‐current density (*j*_L_), electrons transfer number (n) and percentage yield of peroxide.[^32^, ^54^]

**Figure 5:**
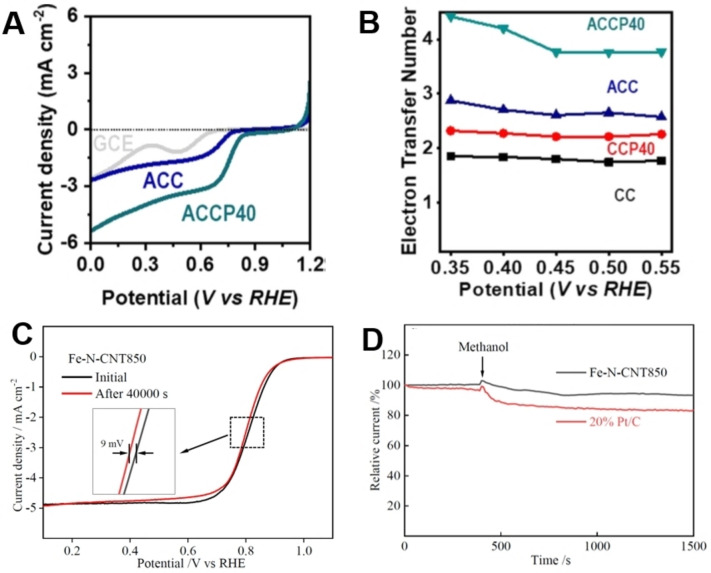
(A) LSV curves for carbon nanofibers derived from waste PVB and cellulose (ACCP40) using a RRDE (2000 rpm) in O_2_‐saturated 0.1 m KOH solution at a scan rate of 2 mV s^−1^. (B) The electron transfer number for ORR at various potentials. Adapted with permission from Ref. [49]; copyright Elsevier 2020. (C) LSV polarization curves of Fe−N−CNT850 obtained before and after stability testing. (D) Chronoamperometric responses of Fe−N−CNT850 and 20 % Pt/C at 0.6 V in O_2_‐saturated 0.1 m KOH with and without methanol. Adapted from Ref. [52]; copyright Wiley‐VCH 2020.

Veksha et al. demonstrated encouraging ORR activity of the MWCNTs derived from flexible packaging waste as a replacement of precious metals and/or expensive carbon‐based nanomaterials.^[57]^ In their study, the key deciding factor towards ORR performance was the appropriate proportion of PET in the pyrolysis feedstock. Lesser PET fractions gave rise to an increase in defect density, which ultimately contributed to the superior ORR activity in 0.1 m KOH electrolyte.^[57]^ Moo et al. observed that besides the PET incorporation in the mixed plastic feedstock, synthesis temperature is another factor affecting the ORR activity of the achieved electrocatalysts.^[68]^ CNTs produced at lower temperatures outperformed the CNTs produced at higher temperatures, owing to numerous edge defects and a lower amount of oxygen‐based surface functionalities. Carbon nanofibers derived from the blend of 40 % waste PVB and cellulose (ACCP40) exhibited promising electrocatalysts with *E*_1/2_ of 0.76 V versus RHE in 0.1 m KOH electrolyte (Figure 5A).^[49]^ Moreover, the ACCP40 nanofibers followed the four‐electron pathway which was mainly attributed to the fibrous morphology, uniformly distributed porosity and highly defective graphitic layers (Figure 5B). In comparison to pure carbon‐based electrocatalysts, heteroatom‐doped carbons are believed to be more efficient for ORR.[^89^, ^100^] During physicochemical evaluation of waste PET‐derived CNTs with nitrogen and iron incorporation, Sridhar and Park noticed the presence of Fe−N_*x*_ moieties within and alongside the walls of CNTs; however, the nitrogen‐carrying moieties were embedded on the outer surface of the bamboo‐like CNTs. Such a combination of evenly distributed and easily accessible active sites enabled a rapid catalytic activity together with enhanced mass transfer rate during ORR, which thereby ensured the remarkable *E*_onset_ of 0.896 V versus RHE.^[70]^

Another critical indicator for the practical performance of electrocatalysts is their long‐term stability in electrolyte and tolerance against methanol crossover, which is primarily important for the direct‐methanol fuel cells (DMFCs).[^98^, ^99^] The biggest obstacle to the practical realization of DMFCs is the lower tolerance of Pt electrocatalysts against methanol crossover that not only shrinks the ORR stability but also limits the overall efficiency of the full device.^[121]^ Hence, an efficient ORR electrocatalyst is supposed to have substantial resistance against methanol poisoning. Cai et al evaluated the methanol tolerance of Fe−N−CNT850 and benchmark catalyst of 20 wt% Pt/C, by recording their respective chronoamperometric response in the O_2_‐saturated methanolic KOH electrolyte (Figure 5C,D).^[52]^ It was reported that 20 wt% Pt/C started deteriorating within the first 400 s; however, Fe−N−CNT850 exhibited much‐prolonged stability in the presence of methanol. The enhanced durability of Fe−N−CNT850 might be attributed to the lower or null interaction and affinity of the iron centers with the methanol.[^52^, ^122^] On the other hand, optimum Fe to Ni ratio (1 : 2) in the bimetallic CNTs led to retained current densities over 40000 s and four‐electrons transformation together with *E*_1/2_ of 0.88 V.^[50]^

It is well‐documented that ORR mechanisms also depend on the pH of the electrolyte, and the switch from acid to alkaline pathway depends on the nature of the electrocatalyst.[^44^, ^104^, ^123^] When the pH of the electrolyte switches from acidic to alkaline, the overall four‐electrons oxygen reduction varies, which modifies the intrinsic activity and durability of catalytic sites within the electrocatalyst.^[124]^ Daniel et al. experienced a definite pH selectivity of waste PU‐derived electrocatalysts towards the four‐electrons and two‐electrons ORR pathways in acidic and alkaline electrolytes, respectively.^[51]^ In the acidic electrolyte (0.5 m H_2_SO_4_), eventual peroxide production was less than 5 %; conversely, it was preferentially increased to 60 % in the alkaline pH region (0.5 m KOH). In these electrocatalysts, Fe−N_*x*_ and pyrrolic nitrogen were the main active sites contributing to both four‐electrons and two‐electrons ORR pathways, respectively. However, in the acidic medium predominance of four‐electrons ORR was fundamentally attributed to well disperse and easily accessible Fe_3_C and α‐Fe particles embedded in the graphite layers. Fe_3_C and α‐Fe acted as co‐catalysts for the rapid reduction of the adsorbed oxygen on the main Fe−N_*x*_ site, shrinking the peroxide formation.^[117]^ However, in an alkaline medium, these Fe‐containing moieties are blocked with the adsorption of ORR intermediates and become inactive.^[88]^ The abundance of pyrrolic nitrogen could be a governing factor in the enhanced selectivity towards peroxide formation in an alkaline medium.^[51]^

**Figure 6:**
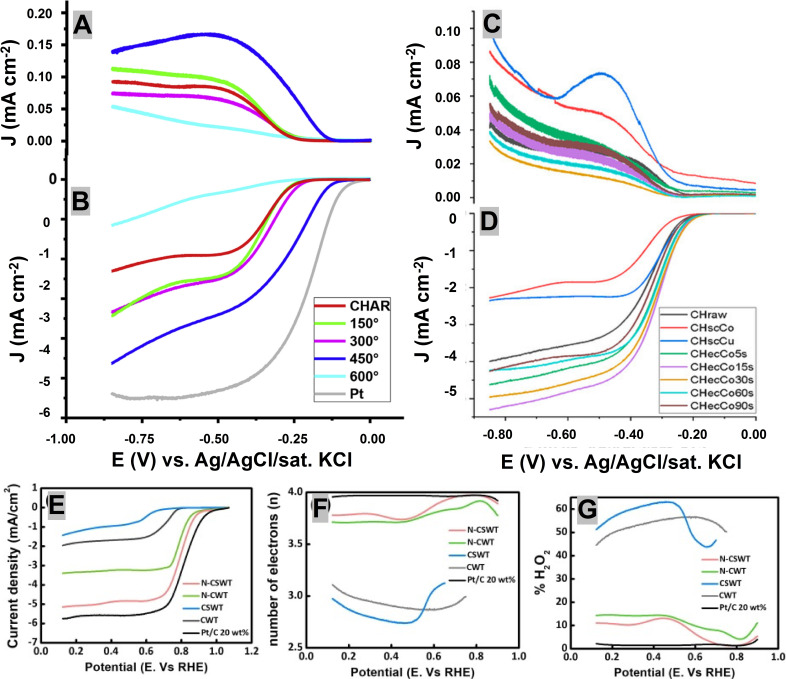
(A,B) RRDE performance including ring current (A) and disk current (B) for waste‐derived char obtained at different temperatures. Adapted with permission from Ref. [85], copyright Elsevier 2020. (C,D) Ring current (C) and disk current (D) for waste‐derived char obtained by using sonochemical and electrochemical deposition of Co and Cu. Adapted with permission from Ref. [86] under CC‐BY‐NC‐ND 4.0 (https://creativecommons.org/licenses/by‐nc‐nd/4.0); MDPI 2020. (E–G) Disk current (E), number of electrons transferred (F), and percentage of peroxide produced (G) from carbonized waste tire (CWT), carbonized sulfonated WT (CSWT), carbonized waste tire (CWT), carbonized waste tire in Ar/NH_3_ atmosphere (N‐CWT), carbonized sulfonated WT in Ar/NH_3_ atmosphere (N‐CSWT), and commercially available Pt/C electrocatalyst. Adapted with permission from Ref. [87] under CC‐BY‐NC‐ND 4.0 (https://creativecommons.org/licenses/by‐nc‐nd/4.0); Elsevier 2021.

Passaponti et al. analyzed the electrocatalytic performance of char derived from scrap tires in alkaline medium (KOH) and found that the char annealed at 450 °C was the best owing to oxygen vacancies induced during the transformation of β‐ZnS into ZnO (Figure 6A,B).^[85]^ The electrocatalytic performance of the char obtained from MAP of waste tires was further strengthened through the electrochemical deposition of cobalt. The cobalt deposition brought a positive shift of 40 mV as compared to untreated‐char.^[86]^ The optimal enrichment conditions (i. e. deposition time of 30 s) guaranteed the electron transfer close to 4.0, peroxide formation under 1 % and maintained stability over the extended period (Figure 6C,D).^[86]^

Kang et al. reported the outstanding ORR activity of nitrogen‐doped, carbon‐based electrocatalysts derived from sulfonated waste tires (N‐WCST), where the *E*_onset_, electron‐transfer number and peroxide formation were approximately 0.89 V, 4.0 and <10 %, respectively (Figure 6E–G).^[87]^ This exceptional performance was consistent with high nitrogen content and the increased electrochemical surface area encompassing abundant active sites. Table 3 compares the ORR performance of various waste‐derived ORR electrocatalysts of the waste plastic polymeric origins.

**Table 3:** Comparison of the ORR performance among various electrocatalysts obtained from plastic waste.

Waste plastic source	Electrolyte	Onset potential [V vs RHE]	Half‐wave potential [V vs RHE]	Current density [mA cm^−2^]	Peroxide formation [%]	Electrons transferred	Ref.
PET packaging	0.1 m KOH	0.931	N/A	0.060	N/A	N/A	[57]
Mixed plastics	0.1 m KOH	0.854	N/A	N/A	N/A	N/A	[68]
PVB	0.1 m KOH	N/A	0.76	N/A	N/A	≈4	[49]
PET bottles	0.1 m KOH	0.896	0.75	4.8	N/A	3.8	[70]
PP lunchboxes	0.1 m KOH	0.943	0.811	N/A	N/A	3.81–3.92	[52]
PP lunchboxes	0.1 m KOH	1.01	0.88	N/A	N/A	3.79–3.98	[50]
Polyurethane	0.5 m H_2_SO_4_	N/A	0.696	3.72	2.0	3.97	[51]
Scrap tires	0.1 m KOH	0.874	N/A	N/A	15.0	3.69	[85]
Scrap tires	0.1 m KOH	0.792	N/A	N/A	1.0	3.98	[86]
Scrap tires	0.1 m KOH	0.89	N/A	N/A	10	≈4	[87]
Scrap tires	1 m KOH	0.832	N/A	N/A	N/A	N/A	[82]

## Conclusions and Perspectives

In this Minireview, the most recent studies related to PGM‐free electrocatalysts for ORR derived from waste plastics were critically summarized. Various plastic wastes have been utilized as precursors for synthesizing ORR electrocatalysts, mainly by transformation through the controlled atmosphere and thermal pyrolysis or microwave‐assisted techniques. As mentioned above, as a large percentage of plastic waste is disposed in landfills without being recycled, reused, or recovered, its transformation in a value‐added products is strongly encouraged. This route is particularly envisioned within the Circular Economy approach in which waste is transformed into a resource. Generally, PGM‐free electrocatalysts are mainly composed of more than 99 % of a carbon backbone, in which defects containing nitrogen and metals coordinated with nitrogen are embedded into the graphitic/graphene‐like structure. Consequently, waste plastics possessing a very high percentage of carbon in their structure seem a logical pathway to pursue to be transformed into carbonaceous materials for application in electrocatalysis, chiefly after functionalization with the transition metal(s) of interest. However, while this route looks encouraging, the electrochemical activity of the PGM‐free materials obtained from these valorization processes is still far from the state‐of‐the‐art PGM‐free electrochemical results obtained in the literature. This can be due to more complex and difficult control of the transformation conditions during pyrolysis or microwave‐assisted processes for which optimization is needed. To the best of our knowledge, only a few manuscripts have been proposed so far aiming at understanding the transformations and functionalization processes for preparing efficient and high‐performing ORR PGM‐free electrocatalysts. The increased efficiency of the ORR electrocatalysts can be achieved through proper engineering of active sites in terms of numerous, atomically dispersed and easily accessible M−N_*x*_−C moieties, pyridinic nitrogen and hydrogenated or protonated edge defects.^[117]^ Consequently, the processing‐structure‐property relationship for the waste‐derived ORR electrocatalysts needs to be established which could provide scientific understandings about the engineering of the active sites.^[125]^ Interdependencies between the electrocatalyst fabrication methodology, surface chemistry and morphological characteristics are critically important to evaluate.

The very first challenge encountered during the transformation of plastic waste into electrocatalysts is the lower yield of carbonaceous char (carbon precursor) obtained from the traditional pyrolysis processes due to generation of oil and gaseous hydrocarbons in higher proportions.^[51]^ Therefore, technological advancements towards the efficient, cost‐effective and scalable pyrolysis of plastic waste are extremely important to provide scientific guidelines for the choice of suitable procedures for particular sources to get higher carbonaceous materials from given sources.^[5]^ The initial preparation conditions for the electrocatalysts such as mixing of different waste plastics percentage, homogenization of the sample, grounding the precursors to a fine powder should be the subject of extended studies. Moreover, process parameters such as pyrolysis temperature, atmosphere, heating and cooling rates must be studied and optimized to obtain a controlled and defined morphology and surface chemistry.[^126^, ^127^] For doping and surface functionalization, the role of precursors choice could be an important research topic.^[128]^ Higher surface area of the electrocatalysts could be achieved using sacrificial templating, chemical activation (wet or dry impregnation) and mechanochemical techniques such as ball milling.^[129–135^] Post‐pyrolysis processes and acid (or basic) washing need also to be further studied and optimized in order to achieve the atomically dispersed (coordinated with nitrogen) and chemically robust active sites.[^51^, ^104^, ^130^] The recent trend was observed from the transformation of waste plastic into metal‐free to transition metal‐based carbon nanomaterials however, the heteroatoms (S, P, B etc.) doping could also be researched to further enhance the activity and selectivity of the electrocatalysts.^[136]^

Although, in the past few years, pyrolysis has emerged as a potential methodology for the fabrication of ORR electrocatalysts, however, deep mechanistic knowledge is still lacking.[^137^, ^138^] Hence, the evolution of structural, morphological, chemical and interfacial modifications during pyrolysis must be investigated with state‐of‐the‐art in‐situ and ex‐situ techniques.^[139]^ Furthermore, the single‐atom electrocatalysts (SAECs), owing to their definite structural coordination, outstanding intrinsic activity and highest possible atomic efficiency, are emerging as new frontiers in the field of electrocatalysis and can potentially substitute the PGM‐based electrocatalysts.[^101^, ^102^, ^140^, ^141^] However, fabrication of SAECs with homogenous and heavily dense active moieties within the carbonaceous framework is still a technical issue. Therefore, future research work addressing the SAECs derived from plastic waste could constitute a novel sub‐field in the domain of PGM‐free ORR electrocatalysis.

**Figure 7:**
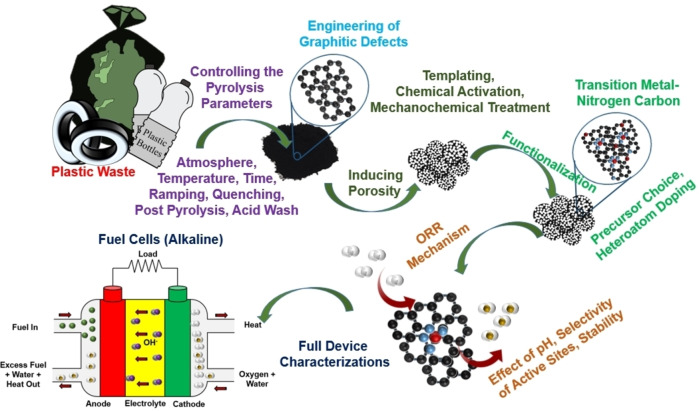
Schematic summary of the prospective research directions in the domain of plastic waste‐derived ORR electrocatalysts.

Traditionally, rotating disk electrode or rotating ring disk electrode examinations are employed as probes for determining intrinsic electrocatalytic activities. However, within this approach, the kinetic behavior of the electrocatalysts is far from the real‐world device applicability since the chief parameters influencing the performance of operating full cells, such as mass transportation, ionic movement, catalyst layer in the membrane electrode assembly (MEA), or water and heat management, are omitted in the half‐cell.[^32^, ^53^, ^105^] Therefore, full‐cell testing in a single‐device configuration is strongly recommended for future developments. Varying the pH of the electrolyte can lead to fundamental changes in the ORR mechanisms. Therefore, the effects of electrolyte pH on the underlying ORR mechanism and activity of the electrocatalysts should also be a part of prospective scientific investigations.^[104]^ Sluggish ORR activities and expensive PGM electrocatalysts are also considered as the main bottleneck in the commercial realization of metal–air batteries, so the waste‐derived electrocatalysts can also find their potential applications in this important domain of advanced energy storage technologies.[^142^, ^143^] The prospective research directions are schematically summarized in Figure 7.

## Conflict of interest

The authors declare no conflict of interest.

## Biographical Information

*Mohsin Muhyuddin obtained his Bachelor of Science degree in Materials Science and Engineering (MS&E) from the Institute of Space Technology (IST), Islamabad, Pakistan in 2016. He completed his Master's in MS&E at IST in 2019. Currently, he is pursuing his doctoral studies at the Department of Materials Science and Nanotechnology at the University of Milano–Bicocca, Italy under the supervision of Dr. Carlo Santoro. His research interests include the development and characterization of low‐cost platinum group metal‐free electrocatalysts and the engineering of nanomaterials for energy‐related applications*.

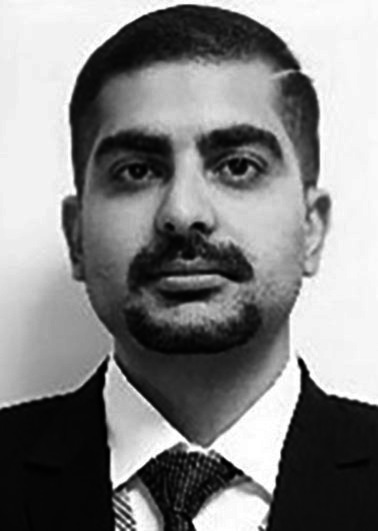

## Biographical Information

*Piercarlo Mustarelli is full professor of Physical Chemistry and Electrochemistry at the Department of Materials Science of the University of Milano–Bicocca. He is also the Chair of the INSTM Italian National Reference Centre for Electrochemical Energy Storage. His research interests span from materials for batteries and fuel cells, to biomaterials, to solid‐state NMR characterization*.

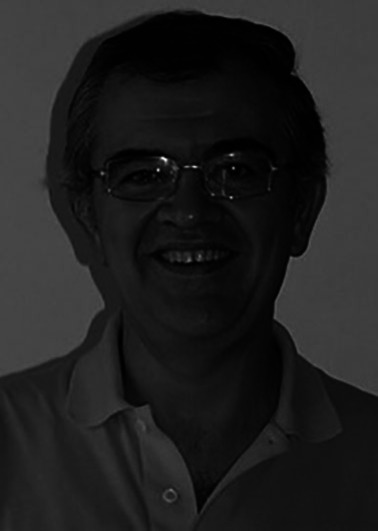

## Biographical Information

*Carlo Santoro is an Environmental Engineer by training (Politecnico di Milano). He obtained his Ph.D. at the University of Connecticut in 2009, working on microbial fuel cells. He moved to the University of New Mexico in 2013 as a postdoctoral researcher working on low‐cost platinum‐free catalyst for oxygen reduction reaction and supercapacitive bio‐electrochemical systems. Following a spell as Lecturer at the University of Manchester in 2020, he joined the University of Milano‐Bicocca as Assistant Professor, where he has established the Electrocatalysis and Bioelectrocatalysis Lab. His work focuses on development of electrocatalysts for based on platinum group metal‐free materials, pursuing biomimetic and bioinspired approaches, and on bioelectrochemical systems (enzymatic and microbial)*.